# Sorghum-Phosphate Solubilizers Interactions: Crop Nutrition, Biotic Stress Alleviation, and Yield Optimization

**DOI:** 10.3389/fpls.2021.746780

**Published:** 2021-12-01

**Authors:** Asfa Rizvi, Bilal Ahmed, Mohammad Saghir Khan, Shahid Umar, Jintae Lee

**Affiliations:** ^1^Department of Botany, School of Chemical and Life Sciences, Jamia Hamdard, New Delhi, India; ^2^School of Chemical Engineering, Yeungnam University, Gyeongsan, South Korea; ^3^Department of Agricultural Microbiology, Faculty of Agricultural Sciences, Aligarh Muslim University, Aligarh, India

**Keywords:** sorghum, phosphate solubilizers, microbiome, phytopathogens, antagonists, P-nutrition

## Abstract

Sweet sorghum [*Sorghum bicolor* (L.) Moench] is a highly productive, gluten-free cereal crop plant that can be used as an alternative energy resource, human food, and livestock feed or for biofuel-ethanol production. Phosphate fertilization is a common practice to optimize sorghum yield but because of high cost, environmental hazards, and soil fertility reduction, the use of chemical P fertilizer is discouraged. Due to this, the impetus to search for an inexpensive and eco-friendly microbiome as an alternative to chemical P biofertilizer has been increased. Microbial formulations, especially phosphate solubilizing microbiome (PSM) either alone or in synergism with other rhizobacteria, modify the soil nutrient pool and augment the growth, P nutrition, and yield of sorghum. The use of PSM in sorghum disease management reduces the dependence on pesticides employed to control the phytopathogens damage. The role of PSM in the sorghum cultivation system is, however, relatively unresearched. In this manuscript, the diversity and the strategies adopted by PSM to expedite sorghum yield are reviewed, including the nutritional importance of sorghum in human health and the mechanism of P solubilization by PSM. Also, the impact of solo or composite inoculations of biological enhancers (PSM) with nitrogen fixers or arbuscular mycorrhizal fungi is explained. The approaches employed by PSM to control sorghum phytopathogens are highlighted. The simultaneous bio-enhancing and biocontrol activity of the PS microbiome provides better options for the replacement of chemical P fertilizers and pesticide application in sustainable sorghum production practices.

## Introduction

The constantly increasing human populations and regularly declining agricultural soils have placed extra pressure on sustainable agriculture systems to eliminate human food hunger worldwide (Díaz-Rodríguez et al., 2020). Globally, P fertilization is a common practice to improve sorghum production (Gemenet et al., 2016), but the intemperate application or unrelenting long-term utilization of chemical-P fertilizers cause environmental pollution and reduce soil quality (Alori et al., 2017; Li et al., 2020). The efficiency of P solubilizer is very low (15–20%) due to its rapid fixation in acidic and alkaline soils, both of which are predominant worldwide (Aziz et al., 2014). Though the majority of global soils contain sufficient reserves of total P, most of it remains inaccessible, and soils thus become deficient in P. So, to maintain sorghum production, P fertilizers are applied regularly. The high cost of chemical-P fertilizer acts as a motivator in the quest for an alternative where naturally-occurring P sources such as rock phosphate (RP) serve as a P reservoir. Regrettably, RP is not easily available to plants in soils with a pH>5.5–6, and farmers thus cannot use RP in sorghum fields (Gurdeep and Reddy, 2015). However, the microbial fertilizers, especially phosphate biofertilizers, augment the P use efficiency (PUE) and thereby improve plant P uptake. Collectively, the phosphate solubilizing microbiome (PSM) represents a group of soil organisms that make P available to plants from both inorganic and organic sources by solubilizing and mineralizing complex P compounds (Zaidi et al., 2009; Chawngthu et al., 2020). Among PSM inhabiting a particular environment, bacteria (Rezakhani et al., 2020), fungi (Elfiati et al., 2021), and actinomycetes (Saif et al., 2014) supply mineral nutrients especially P to various food crops including sorghum growing in different agroclimatic regions worldwide (Abawari et al., 2020; Chen et al., 2021). The PSM intervention in sorghum cultivation practices seems to be an incredibly attractive and highly promising approach to ameliorate sorghum production in P-deficient soil (dos Santos et al., 2017). Apart from supplying P, PSM plays an important role in suppressing the damaging impact of phytopathogens causing severe sorghum yield losses (Gopalakrishnan et al., 2015; Mitra et al., 2020). The PSM-mediated management of P nutrition and sorghum phytopathogens is considered an inexpensive and environmentally friendly biotechnological approach and appears to be a realistic substitute to chemical P fertilizers and hazardous pesticides (Saraf et al., 2014; Kumar et al., 2020). However, due to the competition among/between indigenous/introduced microbiome, soil environment, and other factors, the competence of PSM to survive and colonize in the rhizosphere is greatly challenged (Soumare et al., 2020). Despite this, the PSM, when applied as microbiological formulations in field/greenhouse, has been found to enhance the growth and yield of many food crops (Ahmed et al., 2017; Batool and Iqbal, 2019), including sorghum (Vijayalakshmi et al., 2020), by various direct and indirect mechanisms (Khan et al., 2014; Herrera-Quiterio et al., 2020).

Sorghum, a multipurpose cereal crop belonging to the family Poaceae, ranks fourth in terms of worldwide production after wheat, rice, and corn (Shoemaker and Bransby, 2010; Kumar et al., 2017a). It is typically cultivated in the semi-arid tropics (Almodares and Hadi, 2009) and has several economically important potential uses such as food (grain), feed (grain and biomass), biofuel (ethanol production), fiber (paper), fermentation (methane production), and fertilizer (Hasibuan and Nazir, 2017; Dar et al., 2018). Due to excessive pressure on the production of rice for human consumption, there is an urgent need to search for any nutrition-rich alternative food substitutes for rice to satisfy human food demands. Sweet sorghum in this context can serve as a potential food alternative in many countries (Marles et al., 2018). Different approaches like crop rotation, use of resistant cultivars, and agrochemicals to combat phytopathogens have been adopted to enhance the yield and quality of sweet sorghum, but due to several reasons, especially the cost, technical difficulties, emergence of resistance among pathogens against toxic chemicals, residual toxicity to non-target organisms, or environmental pollution, the implementation of such methods/chemicals is discouraged in sorghum cultivation practices. The utilization of microbiological fertilizers particularly PSM is an alternative option to plant growth and production of sweet sorghum under biotic stress conditions. However, little research has been conducted and very little information is available on the ameliorative role of PSM in relation to the growth and yield of sorghum, particularly under biotic stressed conditions. Acknowledging the importance of human food and lack of information on the role of PSM in sorghum production in high throughput agricultural practices, this review attempts to provide the latest information on the biodiversity and physiological variations of PSM and its importance in sorghum P nutrition, biotic stress alleviation and yield optimization/stability in changing agro-ecosystems worldwide. This review further explains the mechanistic basis of disease suppression and highlights the potential role of single and/or composite PSM formulations in sorghum-soil systems. The understanding of the relationship between the PSM and related rhizobacteria and arbuscular mycorrhizal fungi may help in better management of P fertilization and biotic stress alleviation in sorghum growing in different agrosystems while reducing the risk of chemical pollution.

## Sorghum: Food, Nutritional Composition, and Human Health

Sorghum, which is naturally gluten free, is a major cereal grown as a food and feed crop (Kumar et al., 2017b; Zhao et al., 2019). Sorghum is known by different names, such as great millet and guinea corn in West Africa; kafir corn in South Africa; dura in Sudan; jowar in India, and kaoliang in China. Among sorghum-producing countries, the United States is a major sorghum producer, but only a small fraction of grain is consumed here as human food; instead, it is mainly used as animal fodder. Worldwide, sorghum is consumed in various forms, such as alcoholic and non-alcoholic beverages, baked bread, tortillas, porridges, couscous, gruel, steam-cooked products, expanded snacks, cookies, etc. (Murty and Subramanian, 1982). Sorghum can also be processed into starch, flour, grits, and flakes and is used to produce a wide range of industrial products. Due to its nutritional importance, sorghum is incorporated into the human diet more specifically for people who are intolerant to wheat (Kulamarva et al., 2009). It is consumed mostly in northern China, India, and southern Russia, where about 85% of the crops are consumed directly as human food (Dicko et al., 2006).

Nutritionally, sweet sorghum is quite high (62%) in carbohydrates (Barcelos et al., 2016) and has a higher energy output than sugarcane, sugar beet, corn, and wheat (Dar et al., 2018). Sorghum provides important minerals, vitamins, protein, and micronutrients essential for optimal health, growth, and development (Awika and Rooney, 2004; Kulamarva et al., 2009). Recently (Tasie and Gebreyes, 2020), observed proximate composition values such as moisture, ash, crude fat, crude fiber, and crude protein, and CHO in different sorghum varieties, which varied from 9.66 to 12.94, 1.12 to 2.29, 2.48 to 4.60, 2.17 to 8.59, 8.20 to 16.48, and 67.56 to 76.42, respectively. The highest mineral content in sorghum varieties was (mg/100g): P (368), Na (6), Mg (208), K (314), Ca (67), Fe (14), and Zn (6). The maximum tannin was recorded in Lalo (3,337mg/100g) and Dano (2,474mg/100g). Based on the high mineral value, sorghum varieties such as Miskir, Abshir, ESH-1, Meko-1, Red Swazi, and Karimtams can be considered for food product development. Sorghum also contains various phenolics like flavonoids (Shahidi and Naczk, 1995), which inhibit tumor development (Awika and Rooney, 2004), and antioxidants, which make the grain suitable for producing functional foods. It is a gluten-free cereal, which has importance in the occurrence of Celiac Disease (CD), an immunological response to gluten intolerance. The starches and sugars in sorghum are released more slowly than in other cereals (Léder, 2004) and it is thus beneficial for diabetic persons (Toomey, 1988).

## Why is PSM so Important in Sorghum Plant Systems?

Chemical fertilization (e.g., NPK) in agriculture is documented as a successful practice to manage soil fertility and concurrently to increase the quality and yield of cereal crops (Mojid et al., 2012; Saïdou et al., 2018), including sorghum (Sebnie et al., 2020), in different agro-ecological regions. Among plant nutrients, P is the major nutrient after N and is the second most deficient plant nutrient (Munir et al., 2004). Under a P-deficient environment, plants show altered growth and metabolism, and a reduction from 5 to 15% in crop yield has been reported (Shenoy and Kalagudi, 2005). So, to overcome the low P availability and to allow sorghum plants to grow normally, P fertilizers are used in cultivation practices (Roy and Khandaker, 2010). Sorghum requires high levels of chemical fertilizer for optimal growth, which causes a shift in the soil microbial community (Chu et al., 2007; Wang et al., 2018) and environmental degradation leading to human health problems (Adesemoye and Kloepper, 2009; Kang et al., 2011) These problems accentuate the need for new technologies in sorghum production systems to achieve sustainable production systems. The PSM of plant-growth-promoting microbes is of particular interest as it can, either alone or in combination with other related PGPR/AM-fungi, reduce the adverse impact of chemical fertilizers and simultaneously enhances plants’ tolerance to environmental stress (Bhardwaj et al., 2014). Additionally, the PSM promotes root morphogenesis and gradually increases plant height, stem diameter, number of leaves per plant, leaf area, and yield along with protecting plants from phytopathogens attack (Pandey and Gupta, 2020). Also, P supplied either through solubilization or mineralization by PSM participates in cell division, growth of new tissues and nucleic acid structure, protein synthesis regulation, respiration, signal transduction, macro-molecular biosynthesis, phospholipids, carbon metabolism and a wide range of enzymes, energy transfer, and photosynthesis (Kouas et al., 2005; Hameeda et al., 2008). To fulfill P demands, the PSM has been found to be very useful (Qarni et al., 2021). The PSM-sorghum plant synergy could, therefore, be of great practical importance in the management of P nutrition, biotic stress, and yield optimization. Once developed, the PSM formulation benefits sorghum in several ways due to its ability to (i) release P nutrients slowly and as per the need of plants; (ii) complement other minerals; (iii) supply essential agroactive biological enhancers to plants; and (iv) alleviate biotic stress (Khan et al., 2014; García-Fraile et al., 2015; Yadav and Sarkar, 2019). Broadly, the utilization of microbes mediated rock phosphate solubilization has multiple advantages over conventional chemical P fertilizers in sorghum cultivation practices. These advantages are as follows: (i) the PSM-based formulations are safer than chemical fertilizers; (ii) there is no chance of deposition of either toxic materials or microbiome in the food chain; (iii) the self-replicating ability of microbes evades the requirement for repetitive application; and (iv) they reduce dependence on pesticides. Put together, the PSM along with RP could be an incredibly strong strategy to advance the biological and physicochemical fertility of the soil, which eventually enhances the sorghum production at a low cost. While the literature regarding PGPR interactions with the top three cereals (maize, wheat, and rice) is quite sizeable, a search conducted using the words “PSM” and “sorghum” as keywords revealed very few papers in the scientific literature. Also, when searching PSM and sorghum diseases, very few scientific studies are available. Collectively, the amount of information regarding the interactions between PSM (bacteria/fungi/actinomycetes) and sweet sorghum is limited.

## Overview of PSM: Definition, Biodiversity, and P-Solubilization

**Figure 1:**
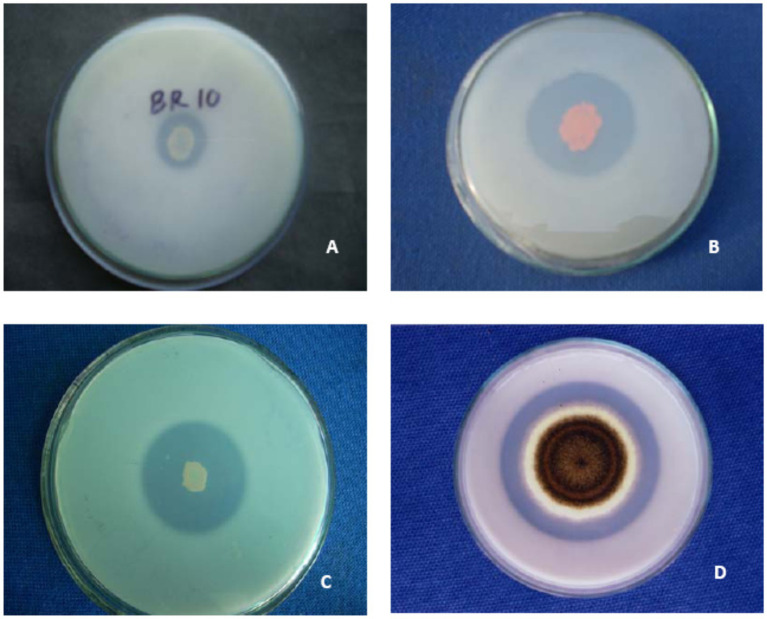
Demonstration of solubilization of insoluble tri-calcium phosphate by species of- **(A)** *Pseudomonas* **(B)** *Serratia* **(C)** *Bacillus* and **(D)** *Aspergillus* on Pikovskaya (1948) medium.

A group of beneficial soil microbiomes, including bacteria, fungi, and actinomycetes, capable of mineralizing/solubilizing complex P into soluble forms are termed phosphate-solubilizing microorganisms (Tian et al., 2021). The PSM has been recovered from different ecological habitats (Table 1) using standard microbiological methods (Figure 1). *Pseudomonas* and *Bacillus* among bacteria and *Aspergillus* and *Penicillium* among fungi are the most efficacious P solubilizers (Li et al., 2016; Zhu et al., 2018). P-solubilizing fungi (PSF) are better P solubilizers than other PSMs due to their ability to do the following: (i) retain a P-dissolving ability after repeated subculturing; (ii) traverse longer distances more easily in any environment; and (iii) produce more organic acids (Venkateswarlu et al., 1984). Among mineral phosphate solubilization (mps) mechanisms, the organic acid (OA) theory is the most widely accepted mechanism of solubilization and supply of P to plants (Table 2). The OA lowers the pH of the environment and causes discharge of P ions from the P mineral by H^+^ exchange for Ca^2+^ (Goldstein et al., 1993). The variation in pH and the amount of P solubilized by PSM has also been unrelated (Asea et al., 1988), which suggests the involvement of mechanisms other than the OA in the P solubilization process which is influenced by many factors (Mohamed et al., 2018). The complex organic P compounds (phosphates, phospholipids, phytin etc.) in contrast are mineralized enzymatically by alkaline and acid phosphatases (Behera et al., 2017; Din et al., 2019), phytases (Din et al., 2019), and phospholipases (Walpola and Yoon, 2012) excreted by PSM.

**Table 1:** Phosphate solubilizing microbiome biodiversity in different agroecological habitat.

PSM groups	Source/Origin	Media used	References
Bacterial genera			
*Enterobacter*	*Capsicum chinense* rhizosphere	PVK	Mendoza-Arroyo et al., 2020
*Pseudomonas* spp.	wheat, barley, maize, oat, faba beans, peas	NBRIP	Elhaissoufi et al., 2020
*Acinetobacter*, *Pseudomonas, Massilia*, *Bacillus*, *Arthrobacter, Stenotrophomonas, Ochrobactrum*, and *Cupriavidus*	Bulk soil	NBRIP	Wan et al., 2020
*Burkholderia cepaciam*, *B. contaminans*	Sweet corn rhizosphere	PVK	Pande et al., 2020
Endophytes			
*Aneurinibacillus* sp. and *Lysinibacillus* sp.	Banana tree roots	NBRIP	Matos et al., 2017
Nitrogen-fixing bacteria			
*Mesorhizobium* spp.	Chickpea root nodules		Muleta et al., 2021
*Rhizobium*, *Agrobacterium*, *Phyllobacterium*	Root nodules of *Acacia cyanophylla*	PVK	Lebrazi et al., 2020
*Mesorhizobium ciceri*, *M. tamadayense*	*Cicer canariense* nodules	NBRIP	Menéndez et al., 2020
*Azotobacter*	Maize rhizospheres	PVK and NBRIP	Nosrati et al., 2014; Bjelić et al., 2015
*Azospirillum* strain	Wheat rhizospheres	PVK, MPVK, and LB	Ayyaz et al., 2016
*A. vinelandii*	Soil	MPVK	El-Badry et al., 2016
P-solubilizing fungi (PSF)			
*Aspergillus hydei*, *Gongronellahydei*, *P. soli,* and *Talaromyces yunnanensis*	*Quercus rubra* rhizosphere	PVK	Doilom et al., 2020
*Trichoderma koningiopsis*	Rice plant	NBRIP	Tandon et al., 2020;
*Penicillium guanacastense*	*Pinus massoniana* rhizosphere	NBRIP	Qiao et al., 2019
Actinomycetes			
*Streptomyces roseocinereus* and *S. natalensis*	Moroccan oat rhizosphere	MPVK	Chouyia et al., 2020

NBRIP, national botanical research institute phosphate medium; PVK, Pikovskaya medium; MPVK, modified Pikovskaya medium; LB, luria bertani.

**Table 2:** Organic acids secreted by PSM.

PSM	Organic acids	References
Phosphate solubilizing bacteria		
*Bacillus*, *Burkholderia, Paenibacillus* sp.	Gluconic, oxalic, citric, tartaric, succinic, formic, and acetic acid	Chawngthu et al., 2020
*Pantoea*, *Pseudomonas*, *Serratia*, and *Enterobacter*	Oxalic, citric, gluconic succinic, and fumaric acids	Rfaki et al., 2020
*Bacillus* sp. strain AZ17	Pyruvic, succinic, fumaric, malic, tartaric, and oxalic acids	Zúñiga-Silgado et al., 2020
*Pseudomonas* sp. strain AZ5, *Bacillus* sp. strain AZ17	Acetic, oxalic and gluconic acids, acetic, citric, and lactic acids	Zaheer et al., 2019
Phosphate solubilizing fungi		
*Rhizopus stolonifer* and *R. oryzae*	Oxalic, lactic, citric, succinic, gluconic, malic, fumaric, acetic acid, and propionic acids	Mohammed, 2020
*Trichoderma* and *Aspergillus*	Oxalic, citric, formic, tartaric, malic, acetic, and citric acids	Li et al., 2016
*Penicillium oxalicum* and *A. niger*	Gluconic acid, oxalic, propionic, and malic acids	Li et al., 2016
Phosphate solubilizing actinomycetes		
*Streptomyces* sp. KP109810, *Streptomyces* sp. CTM396	Gluconic acid	Farhat et al., 2015

### Mechanisms Used by PSM to Facilitate Sorghum Plant Growth

Most of the cultivable soils in the world have insufficient elemental nutrients like P, N, K, and Zn, or these are unavailable to the plants. Also, poor agricultural practices, such as the disproportionate use of fertilizers, lead to undesirable effects such as soil fertility reduction. Indeed, a major part of chemical fertilizers applied to augment sorghum production is wasted further polluting the agroecosystem (Garnett et al., 2009). Below are some of the main mechanisms used by PSM to optimize the use of crop nutrients and help promote sorghum production.

**Figure 2:**
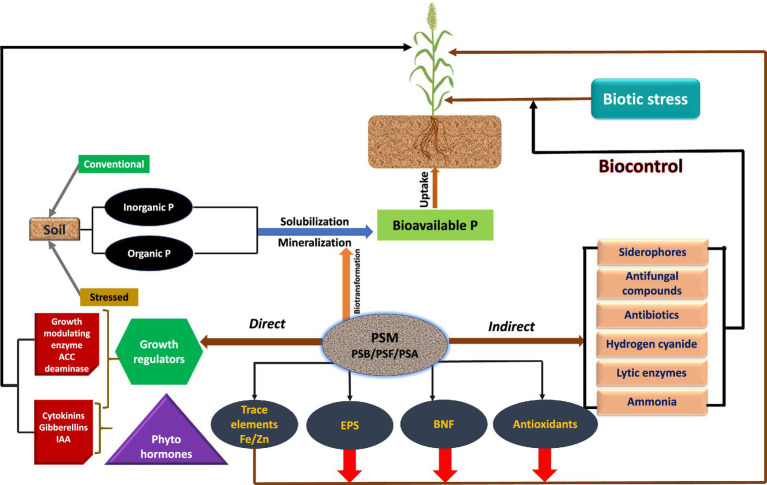
Mechanism used by phosphate solubilizing microbiome (PSM) to facilitate sorghum plant growth.

Apart from supplying inherently P to plants, the P-dissolving microbiome benefits the plants by providing N through BNF, growth modulating enzymes, phytohormones, and antimicrobial compounds, which directly or indirectly affect the growth and development of sorghum (Table 3). The direct growth stimulation by PSM includes the acquisition of nutrients, such as P through solubilization/mineralization and N through N_2_ fixation, phytohormone production, and facilitation of resource while indirectly they promote growth by suppression of plant pathogens and induction of resistance in host plants against pathogens (Figure 2).

**Table 3:** Plant-growth-promoting active biomolecules released by PSM.

Soil microbiome	Source	PGP activities	References
*Mesorhizobium* spp.	Chickpea root nodules	IAA, ACC deaminase, siderophores, HCN	Muleta et al., 2021
*Streptomyces alboviridis* P18–*S. griseorubens* BC3–*S. griseorubens* BC10 and *Nocardiopsis alba* BC11	Desert soils of Morocco	IAA, siderophore, HCN, and ammonia	Boubekri et al., 2021
*Staphylococcus* sp., *Bacillus firmus*, *B. safensis*, and *B. licheniformis*	Soils of rock P mines	IAA, ACC Deaminase	Qarni et al., 2021
*Penicillium* sp. and *Penicillium oxalicum*	Soils of rock P mines	IAA	Qarni et al., 2021
*Burkholderia ubonensis*	Woodland soil of a Chinese fir plantation	IAA, ACC deaminase, nitrogenase, iron carriers	Zhao et al., 2021
*Streptomyces roseocinereus* MS1B15	Moroccan oat rhizosphere	IAA, Siderophores, ACC deaminase, N_2_fixation, antimicrobial activity	Chouyia et al., 2020
*Bacillus* strains	Rhizosphere, leaf endosphere, and sap of P-efficient tropical maize genotypes	IAA	de Sousa et al., 2021
*Agrobacterium tumefaciens* syn. *Rhizobium radiobacter*	Nodules of *Leucaena leucocephala*	Zinc solubilization, IAA, N_2_ fixation, siderophores, EPS, salt tolerance	Verma et al., 2020
*S. roseocinereus, S. natalensis*	Oat rhizospheres	Siderophores, IAA, ACC deaminase, antimicrobial activity against *Fusarium oxysporum*, *Botrytis cinerea*, *Phytophthora cactorum*, and *Phytophthora cryptogea*	Chouyia et al., 2020
*Agrobacterium* sp. NA11001, *Phyllobacterium* sp. C65, *Bacillus* sp. CS14, and *Rhizobium* sp. V3E1.	Root nodules of *Acacia cyanophylla*	IAA	Lebrazi et al., 2020
*Alternaria, Aspergillus, Chaetomium, Curvularia, Fusarium, Melanaspora, Nigrospora, Penicillium* and *Trichoderma*	*Chilli rhizosphere*	IAA, siderophores, HCN, chitinase	Naziya et al., 2020
*Acinetobacter* sp., PGP27, *Ensifer meliloti*	Faba bean and wheat rhizosphere	K solubilization, IAA, EPS	Bechtaoui et al., 2019
*Bacillus* sp.	*Stevia rebaudiana rhizosphere*	IAA, siderophores	Prakash and Arora, 2019
*Lysinibacillus fusiformis*, *Bacillus* sp., *Paenibacillus* sp.	Roots of wheat	IAA,siderophore production, protease activity, antibacterial and antifungal inhibition	Akinrinlola et al., 2018
*Mesorhizobium ciceri* and *M. mediterraneum*	Chickpea nodules	N_2_ fixation, IAA	Zafar et al., 2017
*Klebsiella* sp. Br1, *K. pneumoniae* Fr1, *B. pumilus* S1r1, *Acinetobacter* sp. S3r2	Maize roots	N_2_ fixation,auxin production	Kuan et al., 2016
*Mucror, Penicillium*	*Panax ginseng* rhizosphere	IAA	Hussein and Joo, 2015

The plant growth-promoting substances released by PSM include (i) phytohormones, such as indoleacetic acid (Lebrazi et al., 2020; Qarni et al., 2021) and gibberellin (Pandya et al., 2018; Kang et al., 2019), (ii) asymbiotic (Nosrati et al., 2014; Ayyaz et al., 2016) or symbiotic N_2_ fixation (Muleta et al., 2021), (iii) biocontrol of phytopathogens through antifungal compounds and antibiotics (Olanrewaju and Babalola, 2019; Mitra et al., 2020) or lytic enzymes (Hamane et al., 2020; Naziya et al., 2020), and (iv) secretion of siderophores (Aallam et al., 2021) and HCN (Naziya et al., 2020; Boubekri et al., 2021). In addition to these biological enhancers, the phosphate dissolving bacteria (Kalam et al., 2020; Zhao et al., 2021), actinomycetes (Chukwuneme et al., 2020), and fungi (Ali et al., 2019) also release 1-aminocyclopropane-1-carboxylate (ACC) deaminase to protect plants from attack by pathogens. Phosphate solubilizing microbiota can also protect sorghum plants from biotic stress and stimulate growth indirectly by destructing the metabolism of attacking pathogens and/or stimulate the plant’s immune system. Thus, the indirect mechanisms of PSM could be extremely useful and of great practical interest under field conditions because they provide a chance for growers to avoid the use of chemical biocides (Adesemoye and Kloepper, 2009; Khatoon et al., 2020) and, therefore, protect soils and crops from chemical toxicity.

## PSM-Sorghum Interactions: Inoculation Effects on Growth, Crop Nutrition, and Grain Yield

**Figure 3:**
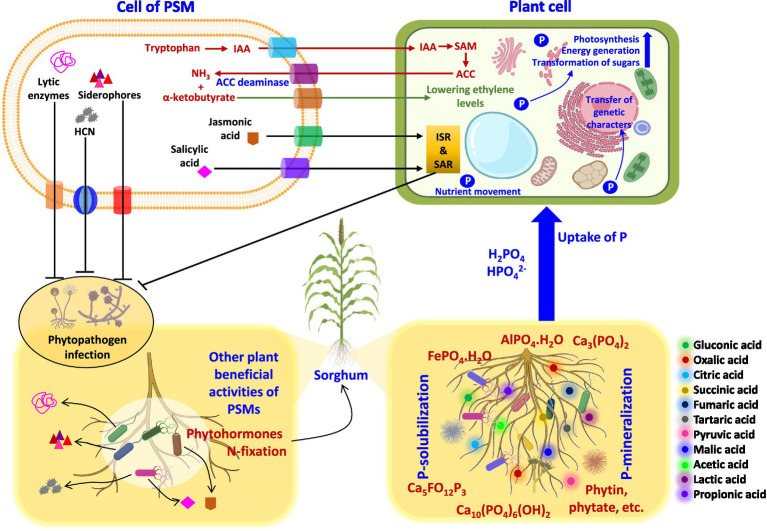
Interactions of PSM with sorghum plants at cellular levels enhancing the growth of sorghum. HCN, hydrogen cyanide; IAA, indole-3-acetic acid; ACC, 1-aminocyclopropane-1-carboxylic acid; SAM, S-adenosyl methionine; ISR, induce systemic resistance; SAR, systemic acquired resistance.

Application of microbes-based bio-fertilizers in agro-ecosystems is beneficial for plant growth and yield enhancement in both conventional (Kalayu, 2019) and extreme environments (Kamali and Mehraban, 2020; Rizvi et al., 2020). Among microbial formulations, the research on PSMs impacting growth, crop nutrition, and yield of cereal crops under greenhouse/field trials are quite large (Table 4). The literature regarding PSM application in sorghum cultivation systems in different agroecological regions is, however, lacking. Considering this gap, the beneficial influence of PSM including PS bacteria (PSB), fungi (PSF), and actinomycetes (PSA) used either alone or in combination with other conventional PGPR or arbuscular mycorrhizal (AM) fungi is highlighted. A model explaining the ameliorative impact of the PSM on morphological, cellular, and physiological activities of the sorghum plant is presented in Figure 3.

**Table 4:** Inoculation effects of PSM on the performance of cereal crops grown in different agroecological systems.

PSM inoculants	Growth parameters of cereals	Experimental conditions	References
Sorghum			
*Bacillus simplex* and *Pseudomonas* sp.	Growth, plant dry matter, and P use efficiency	Greenhouse	Rezakhani et al., 2020
*Aspergillus terreus* and *Penicillium pinophilum*	P uptake and dry matter yield	Greenhouse	Steiner et al., 2016
*Streptomyces* sp.	Growth and yield enhancement	Field	Alekhya and Gopalakrishnan, 2016
Maize			
*Azospirillum brasilense*, *B. subtilis, P. fluorescens*	Improved P uptake efficiency and greater yield	Greenhouse	Pereira et al., 2020
*Achromobacter xylooxidans, Leclerciaa decarboxylata*	Increased photosynthetic rate, stomatal conductance, chlorophyll, carotenoids, and grain yield	Greenhouse	Danish et al., 2020
*B. subtilis*	Increased productivity and shoots P	Field	Lobo et al., 2019
*Aspergillus flavus*	Growth and mineral contents	Greenhouse	Omomowo et al., 2020
*Streptomyces* sp. KP109810	Efficient promotion of maize growth and P content	Greenhouse	Mohammed, 2020
Wheat			
*Streptomyces alboviridis* P18– *S. griseorubens* BC3– *S. griseorubens* BC10 and *Nocardiopsis alba* BC11	Improved root length, root volume, root dry weight, shoot length, and shoot dry weight		Boubekri et al., 2021
*Paenibacillus* sp.	Significantly increased the plant height, biomass, root growth, and P uptake		Chen and Liu, 2019
*Streptomyces*	Plant height, root and shoot dry matter and p uptake	Greenhouse	El-Dsouky et al., 2019
Rice			
*Pantoea* sp.	Significantly increased the plant height, biomass, root growth, and P uptake	Field	Chen and Liu, 2019
*Aspergillus niger*	Increased grain yield	Field	Asuming-Brempong and Anipa, 2014
*Streptomyces* KT 6-4-1	Increased root length, plant height, and dry mass	Greenhouse	Chaiharn et al., 2018
Millet			
*Bacillus* strains	Enhanced biomass production and accumulation of N P K in the shoot	Greenhouse	Ribeiro et al., 2018
*Bacillus* sp. (C2) and *Pseudomonas* sp.	Increased height, total chlorophyll, IAA, starch, fresh and dry weight		Harinathan et al., 2016
Barley			
*Streptomyces roseocinereus* MS1B15	Plant growth and P uptake	Greenhouse	Chouyia et al., 2020

### Inoculation Effects of Single PSM

The inoculation of the P-solubilizing microbiome is considered a promising and inexpensive microbiological option, not to mention the most sustainable one, in food production systems because it increases the P bioavailability without destructing the soil-plant systems. Sorghum, considered a substitute to rice, adapts well to P deficient soils employing absorption and solubilization mechanisms including interaction with PSM. The direct application of RP either alone or/and followed by the inoculation of the PSM is however, a suitable alternative to supply P to the growing sorghum crops. Recently (Mattos et al., 2020), evaluated the effect of PSB inoculation on two sorghum genotypes (BR007-efficient and responsive and SC283-efficient and non-responsive) with different P sources (RP and triple superphosphate, TSP). The sorghum genotypes were inoculated separately with the PSB *Bacillus* strains (B116 and B70) and were cultivated in greenhouse and field soils fertilized with TSP, RP, ½TSP+½RP. The PSB inoculation significantly increased the root biomass and P content under greenhouse and grain yield and grain P content of genotype BR007 grown in the field but had no effect on genotype SC283. The application of PSB as P-bioinoculant with RP acted as a promising alternative to reduce the use of synthetic chemical fertilizers leading to sustainable production of sorghum. The findings of this experiment, however, suggested that the impact of PSB inoculation varied according to (i) sorghum genotype, (ii) P source, and (iii) phosphobacterial strains. A field experiment by (Shete et al., 2018) conducted for three consecutive rabi seasons applying a drought-tolerant PSB strain with or without N and P sources showed a variable effect on the growth and yield of rabi sorghum. The sorghum seed inoculated with PSB strain (Acc. No.1/2012) and 100% recommended N and 75% recommended P_2_O_5_ demonstrated the highest plant height (199.9cm), grain yield (18.13q/ha), stover yield (62.96q/ha), 1,000 grain weight (28.86g), P uptake (14.14kg/ha), benefit: cost ratio (3.17) and PSB counts (4.67×10^4^) at flowering stage. The results firmly indicate a saving of 25% of chemical P fertilizer for rabi sorghum under dryland conditions. Seed inoculation with *Pseudomonas putida* strain 168 in other experiments resulted in the highest dried forage, maximum dry matter digestibility, and crude protein while *P. putida* strain 41 produced maximum water-soluble carbohydrates. Seed inoculation with bacterial strains alone (especially strain 168) increased nutrient uptake, plant growth and consequently enhanced the yield. Among the PSB strains, *P. putida* strain 168 was superior compared even to dual culture application suggesting the antagonism between the two PSB strains (Ehteshami et al., 2014). In a similar investigation, the PSB significantly increased the shoot growth, ear head weight, and P content in both roots and shoots, and the grain yield of sorghum rose under a greenhouse environment, compared to plants grown without bacterial cultures and treated with RP and single superphosphate (Vikram, 2007). The inoculation of PSF, *Aspergillus terreus*, and *Penicillium pinophilum* in association with the reactive phosphate rock (RPR) improved the shoot growth, P-uptake, and enhanced the dry matter yield of sorghum, when grown under greenhouse conditions. The PSF application increased the shoot dry matter yield by 30% while the plant height was enhanced by 42% compared to the uninoculated plants. The PSF inoculation, however, did not affect the root growth of sorghum plants. Moreover, the PSF substantially increased the P concentration by 61 and 71% in the absence and presence of RPR and 110 and 265% in the P accumulation of sorghum relative to the control, respectively. The PSF application, therefore, showed a more obvious beneficial impact on organ growth and sorghum P uptake (Steiner et al., 2016). A study conducted by Srinivasan et al. (2012) revealed that PSB identified using BIOLOG belonging to *Pseudomonas*, *Xanthomonas*, *Bacillus*, *Aerococcus*, *Alteromonas*, *Erwinia*, and *Enterobacter* while PSF identified as *Aspergillus* and *Penicillium* produced IAA and gibberellic acid (GA). The IAA produced by the bacterial cultures ranged between 0.74 and 9.53μg25ml^−1^ while GA varied between 2.08 and 12.55μg 25ml^−1^. The amount of IAA secreted by the PSF differed from 2.33 to 8.69μg 25ml^−1^ and GA ranged from 3.44 to 14.80μg 25ml^−1^. Among the two P-solubilizers, PSF was superior to PSB in terms of P solubilization and increased maximally the stem girth, root length, root dry matter, and total dry matter of sorghum plants.

### Inoculative Synergism Between PSM, N_2_ Fixers, and AM-Fungi

The impact of mixed formulations of bio-enhancers belonging to two or more physiologically related/divergent groups has generally been excellent in promoting crop performance compared to single or mono culture applications (Keller-Pearson et al., 2020; Dierks et al., 2021; Nogales et al., 2021). During composite application, the synergistic or additive effect of the interacting microbe is expected on growth, crop nutrition, and yield of crops. For example, bio-fertilizer organisms can improve the growth of a plant independently through N_2_ fixation or through the production of growth hormones or synergistically through both mechanisms simultaneously. Therefore, a synergistic relationship promotes the growth, crop nutrition, and yield of plants more efficiently than solo culture because the microbial pairing allows the plants to achieve greater absorption of minerals (NPK) and other elements. However, little is known about the synergistic effect of PSM and other PGPR or AM-fungi on sorghum (Cobb et al., 2016). The combined effect of two or more PSM together or PSM with N_2_ fixers or AM fungi on sorghum is reviewed and highlighted.

#### Composite Application of Phosphate Solubilizers

Some recent data by Rezakhani et al. (2020) indicate that the single-and dual cultures of PSB, *Pseudomonas* sp. FA1, and *Bacillus simplex* UT1 and different concentrations of silicon had variable impact on the morphological, nutritional uptake of P, Si and K, and physiological activities of *Sorghum bicolor* plant fertilized with soluble or insoluble P (RP; Rezakhani et al., 2020). In this sense, the RP-fertilized sorghum had better root and shoot biomass while the PSB strains and Si levels applied independently augmented all the measured bio-chemical properties. Application of *Pseudomonas* sp. FA1 and *B. simplex* UT1 and Si with soluble P or insoluble P significantly enhanced P-use efficiency of sorghum plants. As a co-culture, both *B*. *simplex* and *Pseudomonas* sp. considerably increased the dry matter and P uptake of sorghum plants when raised with both forms of P fertilizer. While comparing the inoculation effects of the two PSB, *Pseudomonas* sp. was more efficacious than *B*. *simplex*. The composite culture of PSB with Si had the largest increase in P uptake and other growth indices. The results clearly suggest that the co-application of PSB and Si along with RP fertilization may serve as a substitute for chemical P fertilizer in sustainable sorghum cultivation practices. Studies by (Jisha and Alagawadi, 1996) revealed a variable impact of dual inoculation of PSB bacteria, *P. striata*, or *B. polymyxa* with the cellulolytic fungus, *Trichoderma harzianum*, on the nutrient uptake and yield of *Sorghum* grown in a vertisol treated with cotton stalks. Composite application of *B. polymyxa* or *P. striata* with *T. harzianum* increased the size and weight of the earhead, the number of spikelets per ear, and the straw and grain yield, and N and P uptake significantly relative to uninoculated control and single PSB application. Grain yield was increased by 6–8% due to co-inoculation over mono P-solubilizers inoculation and by 28–30% over *T. harzianum* alone.

#### Synergism Between PSM and N_2_ Fixers

Among phytonutrients, N and P are the two major nutrients that affect many important cellular processes like root elongation, proliferation and changes of root architecture, seed development, and maturity of plants growing both under conventional and stressful conditions (Liu et al., 2020; Patel and Panchal, 2020). The combined inoculation of PSM and N_2_ fixers benefit plants better than either group of organisms used alone while substantially reducing the application of chemical fertilizers under field conditions (Gheliya et al., 2018; Li et al., 2020). Considering this (Afifi et al., 2014), in a field experiment conducted at Ismailia Agricultural Research Station, Agriculture Research Centre (ARC), Egypt, evaluated the impact of an associative N_2_ fixer, *Azospirillum brasilense*, PSB *B*. *megaterium,* and potassium solubilizing bacterium (KSB) *B*. *circulans*, applied either alone or in combined forms and treated individually with either humic or fulvic acids, on the availability of N, P, and K and their overall impact on morphology, physiology, and the yield attributes of *S. bicolor* (cv. Dorado) in the presence of 50% of the recommended dose of mineral NPK. Half of the recommended dose (175kg/fed) of ammonium sulfate (20.5%) was added during sowing and 30 and 60days after sowing with *A*. *brasilense* only. Half of the recommended dose of P (100kg/fed) as super phosphate (15.5% P_2_O_5_) was applied during soil preparation containing *B. megaterium* only, while half of the recommended dose (25kg/fed) of potassium sulfate (48% K_2_O) was applied once only at 60days before the earing stage inoculated with *B. circulans* only. The application of bacterial cultures either alone or as a mixture was more effective and maximally enhanced the growth and the yield of sorghum plants. Among all formulations, the mixture of bacterial cultures and humic acid demonstrated the highest populations of bacterial group in the sorghum rhizosphere. Biological features such as plant height, plant dry weight, and number of branches and biochemical attributes like nitrogenase, dehydrogenase, and phosphatase activities were significantly increased in the presence of co-culture of *A. brasilense*, *B. megaterium*, and *B. circulans* plus humic acid after 45 and 75days of sorghum growth. Also, the NPK, crude protein, and total carbohydrates (%) were at maximum in sorghum grains. The *A. brasilense*, *B. megaterium*, and *B. circulans* when used together exhibited the highest grains yield (3.55 ton/fed) and 1,000 grains weight over control (full NPK). The findings clearly revealed that each bacterial stain could reduce the half doses of NPK through P and K solubilization and N-fixation processes. In a similar recent study, the impact of the asymbiotic nitrogen fixer (*A. chroococcum*) and P solubilizer (*B*. *megaterium*) on growth, yield parameters, and nutrient uptake of *Sorghum bicolor* grown under greenhouse conditions varied considerably (Vijayalakshmi et al., 2020). The formulations consisting of *A. chroococcum* and *B. megaterium* magnified the microbial populations in soil and through synergistic interaction increased the bioavailability of N and P and some other nutrients in the soil during sorghum harvest. The dual culture of N_2_ fixer and P solubilizer applied with a 100% recommended dose of fertilizer (RDF) had a maximum beneficial impact on growth (plant height, length, and dry weight of root and shoot), nutrient uptake (NPK), and grain yield of sorghum compared to the uninoculated plants at harvest. The maximum number of grains (190/plant) and grain weight (5.23g/plant) were observed with 100% RDF and combined formulations of *Azotobacter* with PSB, which was followed by the 75% RDF with *Azotobacter* and PSB. A profound increase in the yield of sorghum was attributed as being largely due to the supply of N and P by the N_2_ fixer and PSB to sorghum crops. This increase was well supported by enhancement in N (highest N 1.9%) and P (highest P uptake 0.41%) uptake by sorghum plants due to inoculation of *Azotobacter* with PSB along with 100% RDF at harvest compared to uninoculated and unfertilized control plants. In a similar greenhouse experiment (Alagawadi and Gaur, 1988, 1992), observed a significant increase in the yield of sorghum due to combined inoculation of PSB, *P. striata* or *B. polymyxa* and N_2_ fixing *A. brasilense* which indicated a positive interaction between the dissimilar groups of bacteria. The yields were, however, markedly further enhanced by the application of 40kgN as urea and 60kg P_2_O_5_ (rock phosphate) per ha along with bacterial cultures over sole application of fertilizer or only bacterial inoculation. Due to this, it was suggested that 40kgN could be saved and the whole quantity of super phosphate could be replaced by RP plus inoculation of *A. brasilense* and *P. striata* or *B. polymyxa*. The population counts of *Azospirillum* and phosphate-solubilizing bacteria in the rhizosphere of sorghum were higher in the respective inoculation treatments than in uninoculated treatments.

#### Synergism Between PSM and AM-Fungi

The AM-fungi during interaction receive sugars, amino acids, vitamins, and other organic substances from the host plant. In return, it absorbs minerals, especially P from the soil, and enhances the growth and yield of the host plant (Johansson et al., 2004). Agronomically, when the two dissimilar groups of beneficial organisms are applied in crop cultivation, the yield of many crops increases substantially (Saxena et al., 2013; Nacoon et al., 2020). Like other PGPR, AM- fungi and PSB interact synergistically wherein PSB solubilize sparingly available P compounds into orthophosphate that AM-fungi absorbs and transport to the host plants (Ordoñez et al., 2016). The reports on the inoculation effects of single or multiple PGPR on many cereal crops are numerous (Wahid et al., 2016; Naseri et al., 2018), but there is a scarcity of information on the synergistic effect of the PSM and AM-fungi on sorghum plants. Below, the role of composite application of PSM and AM fungi in the growth and yield promotion is highlighted.

Principally, AM-fungi provide nutrients to their host plants by producing hyphae that grow out from plant roots, effectively increasing the soil volume from which immobile nutrients can be acquired. So, mycorrhizal agricultural crops perform better and can be more productive compared with non-mycorrhizal plants. In addition, AM-fungi increase and improve the amounts of secondary compounds in plants (Mohamed et al., 2014). The synergy between AM-fungi and PSB with NPK fertilizer demonstrated variable growth and yield impact on greenhouse-grown sweet sorghum under saline conditions (Ramadhani and Widawati, 2020). The dual inoculation of AM-fungi and PSB in the presence of varying doses of NPK increased the plant height, the number of leaves, plant fresh weight, plant dry weight, and P of sweet sorghum plant. The AM-fungi and PSB applied with 25% NPK produced the highest increase in all the biological features of sorghum plants, suggesting that the colonization between AM-fungi and PSB with NPK fertilizer could be a suitable and economical option for enhancing sorghum production even under saline conditions. The results of a study conducted by (Ziaeyan et al., 2016) showed that the plants inoculated with a combination of PSB and AM-fungi expressed synergistic effect to increase the efficiency of P fertilizer and growing conditions and yield of sorghum. The combined application of 25mg P_2_O_5_ per kg of soil along with the PSB and AM-fungi maximally enhanced the biological attributes and therefore, could be recommended to save the P application in sorghum cultivation systems. Nitroxin, a bio-fertilizer prepared from a mixture of asymbiotic N_2_ fixers *Azospirillum* and *Azotobacter* and AM-fungus (*Glomus mosseae*), mitigated the deleterious effects of stress by increasing the amounts of photosynthetic pigments, soluble proteins and osmotic regulation and decreasing electrolyte leakage in sorghum and, hence, increased the yield significantly. Mechanistically, the interacting bacterial genera had N_2_ fixation, P solubilization, and iron releasing abilities, as well as the capability to secret plant hormones such as auxin, cytokinin, and gibberellin and a growth modulating enzyme, ACC-deaminase, which together enhanced the yield substantially (Olanrewaju et al., 2017).

The interactions between PSM, N_2_ fixers, and AM-fungi in terms of facilitating plant growth, nutrient uptake, and yield have been reported in numerous studies in the literature. The information regarding the inoculative effect of PSM, N_2_ fixers AM fungi on sorghum is scanty. Widada et al. (2007) demonstrated that sorghum plants inoculated with PSB (*Pseudomonas* sp.), N_2_ fixer (*Azospirillum lipoferum*), and AM-fungi (*Glomus manihotis* and *Entrophospora colombiana*) and grown in acid and P deficient soils improved the plant dry weight and nutrients (such as N, P, Fe, and Zn) uptake, which were enhanced further by dual inoculation of selected microbiome compared to the single inoculation. Dual inoculation of AM-fungi with PSB and AM-fungi and N_2_ fixer increased plant dry weight by 112 and 64 times compared to the uninoculated plant, respectively. The selected rhizobacteria also improved plant colonization by AM-fungi. These results suggest that the tripartite interaction between PSB, N_2_ fixer, and AM-fungi can be an excellent strategy for optimizing sorghum production since no antagonisms among interacting organisms were observed.

## Biotic Stress Alleviation by PSM

Pathogenic microorganisms are a major and continuing threat to food production and ecosystem stability worldwide. Yield, grain quality, and production stability of sorghum are constrained by various biotic (Kumar and Ram, 2014) and abiotic (Tari et al., 2013) stressors, resulting in poor marketability and utilization and causing severe economic losses. Among biotic factors, numerous fungal and bacterial diseases (Table 5) are of prime concern in sorghum-producing areas across the world. Attempts have been made to reduce pesticide use, develop stress-resistant varieties, enhance ecological fitness, and create a sustainable production system in order to enhance efficiency, grain quality, and profitability (Sharma, 1985; Kumar et al., 2014). None of these approaches have, however, been completely successful due to several reasons such as the production cost, lethal impacts of agrochemicals, emergence of resistance among pathogens toward one or multiple biocides/pesticides, and lack of genetic resistance to sorghum varieties. These factors have generated interest among sorghum growers in employing biotechnological options, including the use of inexpensive PSM in the management of sorghum phytopathogens (Khasa, 2017; Nepomuceno et al., 2019; Mitra et al., 2020). The application of PSM in the abatement of phytopathogens has certain advantages, such as (i) not causing environmental hazards; (ii) precluding residual toxicity; and (iii) blocking the emergence of resistance among pathogens attacking sorghum. PSM controls the pathogen damage by one or simultaneous mechanisms through biocide compounds and optimizes the yields (Zaidi et al., 2014; Choudhary et al., 2016). The literature on the role of PSM in the management of phytopathogens affecting sorghum is, however, without doubt, lacking.

**Table 5:** An overview of various diseases caused in sorghum following biotic stress.

Diseases	Causal organism	Major organs affected	Hotspot locations	References
Bacterial diseases				
Bacterial leaf streak	*Xanthomonas campestris* pv. Holcicola	Leaves	Wide geographical distribution	Wang et al., 2021
Bacterial leaf stripe	*Burkholderia andropogonis*	Leaves, flower buds, peduncles	Semi-arid and tropical regions	Mareya et al., 2020
Bacterial leaf spot	*Pseudomonas syringae*	Leaves	Argentina, Bulgaria, China, Hungary, India, Italy, Mexico, Africa, Rumania, Yugoslavia, Venezuela	Hernandez et al., 1992
Fungal diseases				
Anthracnose	*Colletotrichum sublineolum*	All above-ground parts	United States, India, Mexico, Nigeria	Abreha et al., 2021
Charcoal rot	*Macrophomina phaseolina* (Tassi) Goid	Root and stalk	India, Africa, Australia, United States	Mahmoud et al., 2018
Downy mildew	*Peronosclerospora sorghi*	Seedling and leaves	Africa, Asia, Mexico, America	Prom et al., 2015
Covered kernel smut	*Sporisorium sorghi*	Kernels	Ethiopia, Africa	Nigusie and Ademe, 2020
Head smut	*Sporisorium holci-sorghi*	Panicle	Africa, Europe, North and South America, Mexico, Asia, Australia, New Zealand	Wagari, 2019
Loose kernel smut	*Sporisorium cruentum*	Kernels and panicles	Egypt	Moharam, 2018
Rust	*Puccinia purpurea*	Leaves	Nigeria, India, Mexico, United States	Wang et al., 2014
Rough leaf spot	*Ascochyta sorghi*	Leaves	China	Xu et al., 2019
Leaf blight	*Helminthosporium turcicum*	Leaves	Mexico, Brazil, India, Sudan, Nigeria, Niger, Kenya, and Ethiopia	Das and Rajendrakumar, 2016
Ergot	*Claviceps sorghi*	Spikelets	Israel	Shimshoni et al., 2017
Pokkah Boeng (twisted top)	*Fusarium subglutinans*	Leaf and top	India	Das et al., 2019
Zonate leaf spot	*Gloeocercospora sorghi*	Leaves	China	Jiang et al., 2018
Sooty stripe	*Ramulispora sorghi*	Leaves	United States	Brady et al., 2011
Grain mold	*Aspergillus* sp., *Penicillium* sp.	Grains	Asia and Africa	Das et al., 2020
Target leaf spot	*Bipolaris sorghicola*	Leaves	United States, India, Japan	Kimball et al., 2019

### Mechanism of Disease Suppression

The PSM controls the phytopathogens attack by synthesizing pivotal molecules like siderophores (Sarr et al., 2020), antibiotics (Raaijmakers and Mazzola, 2012), lytic enzymes (Herrera-Quiterio et al., 2020), cyanogenic compounds (Munir et al., 2019), and through induction of systemic resistance (ISR; Audenaert et al., 2002). Due to this, the soil PSM improves the growth and grain yield of sorghum by suppressing the damaging impact of biotic stresses. Below are some of the main mechanisms employed by PSM to manage phytopathogens and to boost sorghum yields.

#### Siderophores

Under iron-starved conditions, numerous PSMs, including bacteria, fungi, and actinomycetes, produce a wide range of siderophores (Maldonado et al., 2020; Aallam et al., 2021) with relatively low molecular weight (below ≈2kDa) and ferric ion-specific chelating agents at neutral to alkaline pH to solubilize, capture, and transport inorganic iron to cell (Sandy and Butler, 2009). They are, therefore, exploited in the management of plant diseases (Verma et al., 2011; Jyothi et al., 2020). The siderophore positive rhizobacteria protect the plants from damages by preventing the iron acquisition (Estrella and Chet, 1998) and, hence, limiting the proliferation and root colonization by phytopathogens (O’sullivan and O’Gara, 1992).

Charcoal rot of sorghum caused by *Macrophomina phaseolina*, a soil-and seed-borne disease of sorghum, is endemic to tropical and temperate regions of the world (Kumar et al., 2016). Charcoal root causes considerable yield losses due to a lack of genetic-resistant sorghum varieties. Up to 64% yield losses are reported in India by this disease during the post-rainy season (Das et al., 2008). In order to control the attack of this phytopathogen (Gopalakrishnan et al., 2011), isolated siderophore-positive PSB, *Pseudomonas plecoglossicida*, *B. altitudinis*, *E. ludwigii*, *Acinetobacter tandoii*, and *P. monteilii* from the rhizospheres of a system of rice intensification (SRI) fields and tested their biocontrol activity against *M. phaseolina*. Interestingly, all PSB showed strong antagonistic activity in dual culture assay, blotter paper assay, and *in vivo* greenhouse and inhibited the growth of *M. phaseolina*. No charcoal rot infection was observed in *P. plecoglossicida*-treated sorghum roots indicating the complete inhibition of pathogen whereas the roots treated with other isolates had 49–76% less charcoal rot infection compared to the control. All PSB increased root and shoot dry mass by 15–20% and 15–23% over control under greenhouse experiment. Also, all PSB adapted well to the sorghum rhizosphere as indicated by the reduction in charcoal rot disease. Similar antifungal activities (inhibition of growth, biomass, microsclerotia production, spore germination) of the secondary metabolites (antifungal volatile and siderophores) and the cell-free culture filtrates (CFCFs) of the selected fluorescent pseudomonad strains (SRB129, SRB288, and *Pseudomonas chlororaphis* SRB127) against the mycelial growth of *M. phaseolina* that varied from 30.5 to 76.5% in dual culture assay is reported (Das et al., 2008) The CFCF of the fluorescent pseudomonads @ 20% (v/v) significantly decreased the formation and germination of microsclerotia of *M. phaseolina*. Siderophore-positive bacterium *P. chlororaphis* (SRB127) controlled the charcoal rot of sorghum most efficiently under field conditions. As seed treatment, the bacterium reduced the charcoal rot incidence by >40% and crop-lodging by >20% and increased the sorghum grain mass. Under glasshouse conditions, the bacterium survived in the sorghum rhizosphere without any significant reduction in its population. Similarly, *Streptomyces* isolated from medicinal plant rhizosphere inhibited the fungal activity of phytopathogenic fungi *A. brassicicola*, *C. gloeosporioides*, *Fusarium oxysporum*, *P. digitatum*, and *S. rolfsii* through siderophores (Khamna et al., 2009). de Los Santos-Villalobos et al. (2012) reported the inhibition of *Colletotrichum* species (causing anthracnose in sorghum) due to the release of hydroxamate siderophore by *B. cepacia* in a Petri-dish bioassay test. Interestingly, even the lowest concentration (0.64μgml^−1^) of siderophore inhibited the pathogen by 91%. Other phytopathogenic fungi capable of inflicting damage to sorghum for example, grain mold diseases of sorghum (*Aspergillus* and *Penicillium*) has also been controlled by siderophore rich culture and free supernatant of *Alcaligenes* sp., and *P. aeruginosa* (Sayyed and Patel, 2011). The siderophore based biological control measures, therefore, be adopted in sorghum cultivation practices due to reasons, as they are (i) inexpensive and non-destructive (safer) for the environment; (ii) self-replicating and do not require repeat application; and (iii) there is no emergence of resistance among target organisms (Sayyed et al., 2004).

#### Growth Modulating Enzyme

Production of 1-aminocyclopropane-1-carboxylate (ACC) deaminase by PSM is considered yet another most powerful management biological weapon for plants growing under abiotic stress (Glick, 2014). After infection by phytopathogens, ethylene, a stress hormone is produced in all higher plants, which causes senescence, chlorosis, abscission, and wilting in plants, worsening the detrimental impact of pathogens (Dubois et al., 2018; Sharma et al., 2019; Etesami and Glick, 2020). The damaging impact of stress hormone can be annulled by the ACC deaminase, a multimeric enzyme, secreted by PSM bacteria (Zhao et al., 2021), fungi (Aban et al., 2017), and actinomycetes (Chukwuneme et al., 2020), which cleaves ACC (precursor of ethylene) to produce α-ketobutyrate and ammonia and thereby decreases the ethylene levels in host plants (Gupta and Pandey, 2019; Orozco-Mosqueda et al., 2021). The decrease in the levels of ACC and ethylene prevents the ethylene-mediated plant growth inhibition. PSM endowed with this capability can benefit the sorghum plants by reducing the stress and increasing plant growth (Santana et al., 2020). As an example, (Mareque et al., 2015) reported that the majority of the PSB, such as *Pantoea, Enterobacter, Serratia, Pseudomonas, Acinetobacter*, and *Stenotrophomonas* (Gammaproteobacteria phylum), *Achromobacter*, *Herbaspirillum*, and *Ralstonia* (Betaproteobacteria), *Rhizobium* (Alphaproteobacteria), *Chryseobacterium* (Bacterioidetes), *Bacillus, Staphylococcus*, *Brevibacillus,* and *Paenibacillus* (Firmicutes), and *Kocuria* (Actinobacteria), isolated from roots and stems of sweet sorghum plants exhibited ACC deaminase activity. Of these, ACC deaminase positive *Rhizobium* sp. UYSB13 and PSB bacterium *Pantoea* sp. UYSB45 showed a significant difference from the negative control in the stem height and dry weight (roots and shoots). Additionally, the ACC deaminase positive *Rhizobium* sp. UYSB12 and *Enterobacter* sp. UYSB34 significantly enhanced the root and the shoot dry weights of sweet sorghum.

#### Antimicrobials

The antibiotic-mediated inhibition of plant pathogens by rhizosphere-inhabiting biocontrol microorganisms is well (Babalola, 2010; Raaijmakers and Mazzola, 2012) are the most common and have been used in disease management through antibiotics, for example, the suppression of take all disease in wheat by 2,4-diacetylphloroglucinol (Weller et al., 2007). A variety of antibiotics have been identified: amphisin, 2,4-diacetylphloroglucinol (DAPG), oomycin A, phenazine, pyoluteorin, pyrrolnitrin, tensin, tropolone, and cyclic lipopeptides produced by pseudomonads (Raaijmakers and Mazzola, 2012; Saraf et al., 2014), and oligomycin A, kanosamine, zwittermicin A, and xanthobaccin produced by *Bacillus*, *Streptomyces*, and (Milner et al., 1996; Hashidoko et al., 1999; Mavrodi et al., 2012) diffusible products like 2, 4-diacetylphloroglucinol, phenazines, pyoluteorin, pyrrolnitrin, etc., or volatile compounds such as dimethyl disulfide, cyanogenic compounds, etc. (Olanrewaju and Babalola, 2019; Pandey and Gupta, 2020). One problem in depending too much on PSM-based antibiotics as biocontrol agents is, however, that with the increased use of PSM, phytopathogens may also develop resistance to microbe-mediated antibiotics in a manner similar to those exhibited for conventional antibiotics. This can though, be obviated by incorporating an HCN-positive PSM (Mahdi et al., 2020) with antibiotics producing PSM (Hamdali et al., 2008) so that the HCN-positive strain kills the pathogens while avoiding the emergence of antibiotic resistance among phytopathogens. The combination of HCN and antibiotics positive PSM looks a promising strategy that will synergistically control the phytopathogen attack.

#### Hydrolytic Enzymes

A number of hydrolytic enzymes, such as, cellulases, chitinase, β-1,3-glucanase, protease, lipase, and peroxidase, are released by the PSM (Liu et al., 2016; Nandimath et al., 2017). The cell wall of plant pathogenic fungi, for instance *F. oxysporum*, is mainly composed of β-1,3-glucan layers that are highly susceptible to lysis by β-1,3-glucanase. Following degradation, there occurs the leakage of inner cellular contents to the exterior environment and, finally, due to the osmotic imbalance, the pathogenic fungi collapses (Singh et al., 1999). For instance, some PSFs, such as *Acrocalymma* sp., *otryobambusa fusicoccum,* and *Phoma* sp., produce lytic enzymes such as chitinases and glucanases (Silveira et al., 2021) and catalase and cellulases (Amin et al., 2020), and many PS actinomycetes such as *S. fulvissimus*, *Streptoverticillium olivoverticillatum*, *S. nogalater*, *S. longisporoflavus,* and *S. cellulosae* produce cellulase, chitinase, pectinase, lipase, and amylase (Nandimath et al., 2017), which lyse the cell walls of pathogenic fungi attacking sorghum. Similarly, number of PSB belonging to the genera *Chrysobacterium, Bacillus, Pseudomonas, Mycobacterium, Staphylococcus, Curtobacterium, Enterobacter, Agrobacterium, Ochrobactrum, Serratia, Stenotrophomonas,* and *Acinetobacter* produced lytic enzymes, such as proteases, celluloses, lipases, esterases, and amylases, which exhibited activity against *Fusarium*, *Aspergillus*, and *Colletotrichum* (Herrera-Quiterio et al., 2020). In a similar study, phosphate solubilizing *B. subtilis* BN1 isolated from the chirpine (*Pinus roxburghii*) rhizosphere exhibited strong antagonistic activity resulting in vacuolation, hyphal squeezing, swelling, abnormal branching, and lysis of *M. phaseolina*, *F. oxysporum,* and *R. solani*. The inhibition of fungal growth by *B. subtilis* BN1 was attributed due to the secretion of lytic enzymes, chitinase, and β-1,3-glucanase, which degrades the hyphae and digest the fungal cell wall (Singh et al., 2008). The CFCF of BN1 also showed a strong but concentration-dependent antifungal activity and completely inhibited the fungal growth at 60% CFCF concentration. *Pseudomonas putida* in yet another experiment demonstrated antifungal activity against *A. alternata, F. oxysporum, and R. solani* through chitinase, β-1,3 glucanase, salicylic acid, siderophore, and HCN (Selvakumar et al., 2009). The PS bacterium *Serratia marcescens* inhibited the growth of *Sclerotium rolfsii* (Ordentlich et al., 1988) while *Paenibacillus* sp. strain 300 and actinomycetes *Streptomyces* sp. strain 385 suppressed *F. oxysporum* fsp. *Cucumerinum*. Extracellular chitinase and laminarinase synthesized by *Pseudomonas stutzeri* degraded the mycelia of *F. solani* (Lim et al., 1991). These and other related studies that are not included in this review suggests that, in the absence of high level of genetic resistance in high-yielding sorghum varieties, the PSM as bio-antagonists could safely be used to effectively manage the biotic stresses of sorghum and hence to reduce losses in yield and quality of sorghum cultivated in different regions of the world.

## Conclusion

The unwarranted and imprudent application of agrochemicals in sorghum cultivation practices and their hazardous impact on the soil environment warrants the use of a low-cost and environmentally friendly P-solubilizing microbiome as a prospective alternative to P fertilizers and pesticides. Due to the exorbitant cost of chemical fertilizers, harmful impact of pesticides, and lack of high level of genetic resistance, the novel PSM discovered so far and discussed herein applied either alone or in synergism with compatible organisms can be a useful component in the management of P fertilization, yield optimization, and integrated sorghum disease management. The application of PSM-based (microphos) formulations in sorghum cultivation is likely to provide economic benefit to sorghum growers while reducing the risk of environmental pollution.

### Future Prospects

The studies surveyed and presented herein are though directly relevant to improving food production, deciphering the detailed molecular and ecological relationships between PSM, N_2_ fixers, and AM-fungi is indispensable to developing a better understanding of the synergistic relationship between three functionally divergent groups of the soil microbiome. Also, the rhizosphere competence and colonization effect of different PSM strains and their interaction with functionally unrelated plant beneficial bacteria should further be investigated under complex and variable natural conditions with the aim of producing “microphos: PSM based biofertilizer” to alleviate the biotic stresses and consequently augmenting sweet sorghum production in different agroecosystems. Continued research is, however, needed to develop novel strategies to ameliorate the yield and upgrade the efficiency of PSM to manage the attack of phytopathogens causing huge losses to sweet sorghum in variable agroclimatic conditions. To achieve this, scientists, institutions, and manufacturers need to work together to find solutions to nutrition and disease management and to optimize sorghum yield to eradicate hunger worldwide.

## Author Contributions

All authors listed have made substantial, direct and intellectual contributions to the work, and approved it for publication.

## Funding

This work was supported by the Priority Research Centers Program through the National Research Foundation of Korea (NRF) funded by the Ministry of Education (2014R1A6A1031189) to JL and also supported by the NRF grant funded by the Korean government (MSIT; 2021R1G1A1094698) to BA.

## Conflict of Interest

The authors declare that the research was conducted in the absence of any commercial or financial relationships that could be construed as a potential conflict of interest.

## Publisher’s Note

All claims expressed in this article are solely those of the authors and do not necessarily represent those of their affiliated organizations, or those of the publisher, the editors and the reviewers. Any product that may be evaluated in this article, or claim that may be made by its manufacturer, is not guaranteed or endorsed by the publisher.
